# Supplementary material for: Near-Infrared Emission of HgTe Nanoplatelets Tuned by Pb-Doping
Source: Nanomaterials (Basel). 2022 Nov 25;12(23):4198. doi: 10.3390/nano12234198 (PMC9740587; doi:10.3390/nano12234198)
Supplement: Supplementary file 1 [file nanomaterials-12-04198-s001.zip › nanomaterials-2050337-supplementary.pdf]

nanomaterials

Supplementary Information

Near-Infrared Emission of HgTe Nanoplatelets Tuned by Pb-Doping

Chemicals

Mercury(II) acetate ($Hg(OAc)_2$, 98%), tri-n-octylphosphine (TOP, 97%), cadmium acetate dihydrate ($Cd(OAc)_2{\cdot}2H_2O$, 99.99%), lead(II) thiocyanate ($Pb(SCN)_2$, 99.5%), tellurium powder (Te, mesh 200, 99.99%), trioctylamine (TOA, 98%), 1-octadecene (ODE, 90%), oleic acid (OlAc, 90%), and n-hexane (≥95%), isopropanol (HPLC grade) and toluene (≥95%) were purchased from Sigma-Aldrich. All reagents were used as received without further purification.

Synthesis of 3MLs CdTe Nanoplatelts

The synthesis of CdTe NPls followed the procedure published by Ithurria et al [1]. ml of octadecene (ODE) was mixed with 160 mg of cadmium acetate dihydrate ($Cd(Ac)_2{\cdot}2H_2O$) and 306 µl of oleic acid (OA). The solution was degassed at 100 ° C for 1h, after that, flask was switched to the inert atmosphere and quickly heated to 195 ° C. Then, a mixture of 100 µl of 2.24 M complex of trioctylphosphine-tellurium (TOP-Te) and 4.9 ml of ODE was continuously injected into the flask for 15 minutes. The reaction mixture was kept for an additional 15 minutes at this temperature, then cooled down to 150 ° C and 2 ml of OA was added. Then, the vessel was cooled to room temperature, and the reaction product was centrifuged with isopropyl alcohol as antisolvent. Obtained CdTe NPLs were redissolved in 2 ml of hexane for subsequent use.

Synthesis of HgTe Nanoplatelets

The cation exchange procedure proposed by Izquierdo et al. [2] was slightly modified. Briefly, 150 µL of the initial solution of CdTe nanoplatelets were added into centrifuge tubes with 12 ml of hexane. Obtained CdTe NPLs solution was mixed with 350 µl of mercury acetate in trioctylamine (concentration 8 mg/ml) and left under vigorous stirring for 18 minutes in a vortex. During the first 2 minutes of the cation exchange process, the color of the solution changed from orange to dark brown. After the end of the substitution, 300 µl of oleic acid was added with additional mixing for another 5 minutes. Exchanged HgTe nanoplatelets were centrifuged at 5000 rpm for 5 minutes and redissolved in toluene.

Determination of the Pb/Hg molar ratio used during doping procedure of HgTe NPLs

Pb/Hg ratio was determined gravimetrically. Briefly, 1ml of HgTe nanoplatelets were taken and purified several times with isopropanol. After that, the precipitation was dried under vaccum and weighted. The amount of HgTe (in moles) was determined with consideration of the fact that Hg:Te molar ratio is equal to Cd:Te molar ratio from initial nanoplatelets. The latter proportion was obtained according to the literature [3].

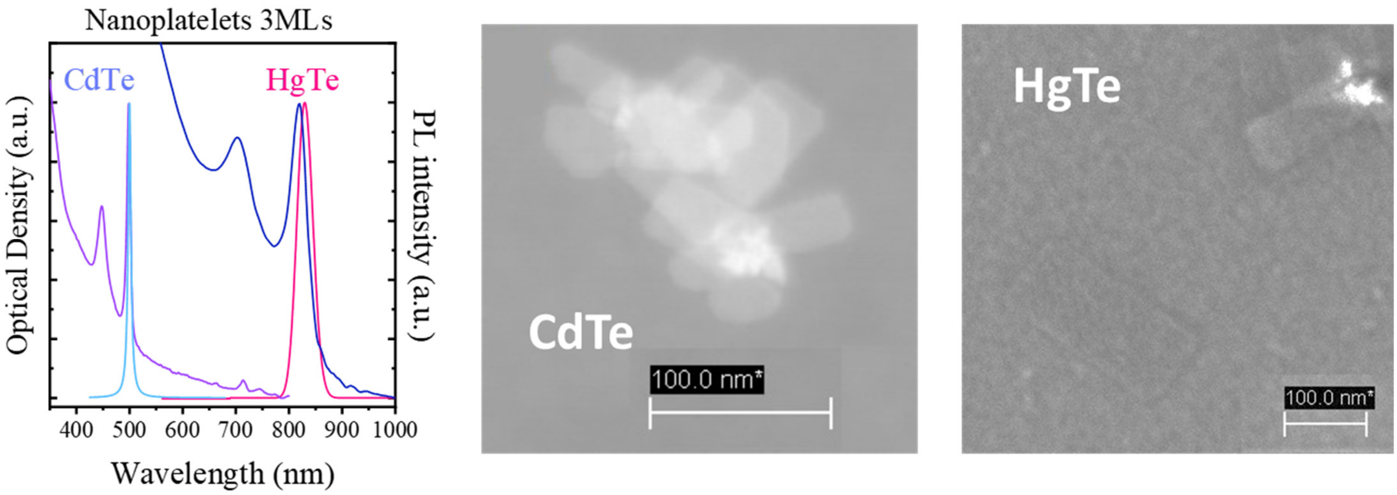

Figure S1. Photoluminescence and absorption spectra of CdTe and HgTe nanoplatelets; SEM image of the initial CdTe NPls; TEM image of the HgTe NPls.

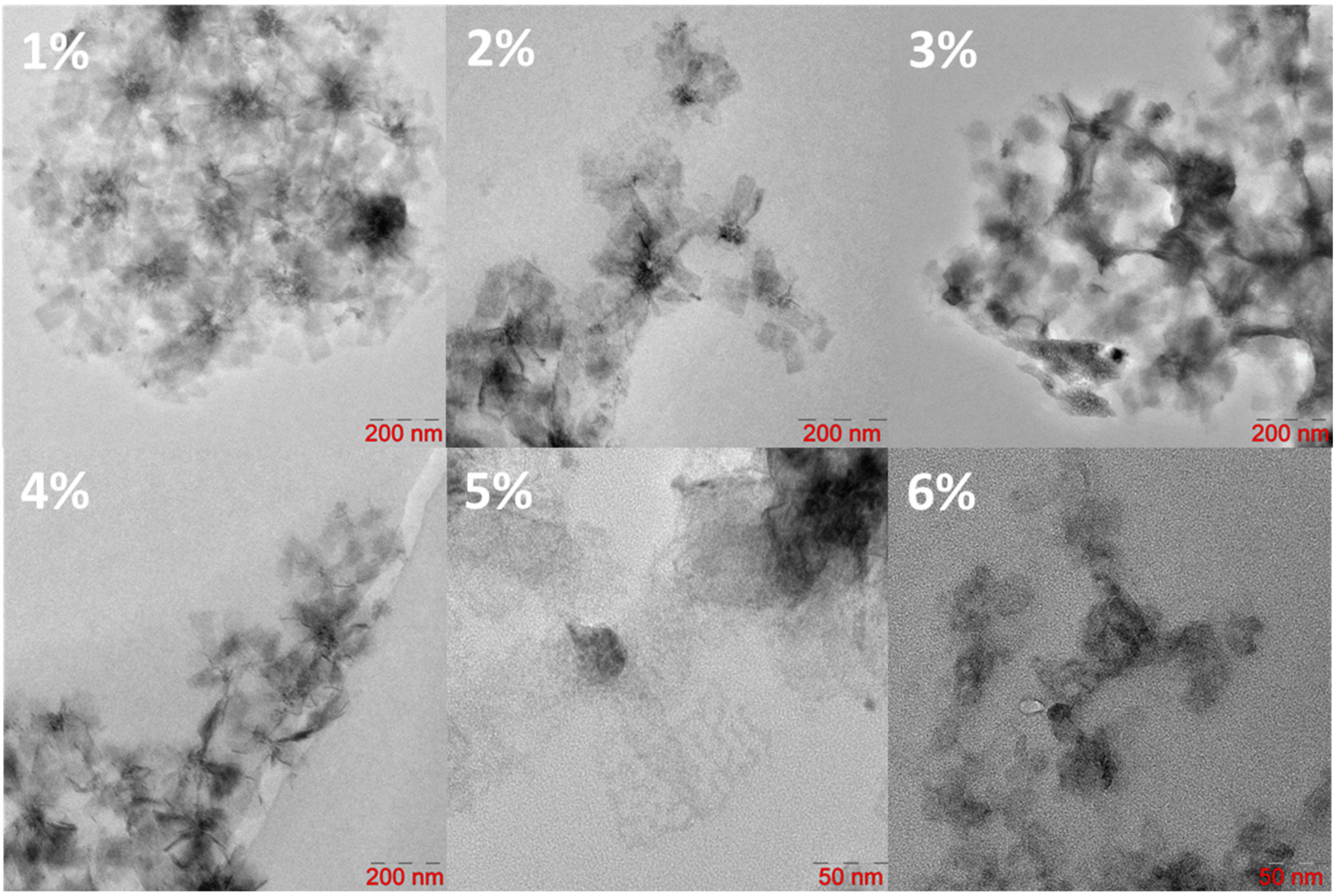

Figure S2. TEM images of HgTe:Pb NPLs with 1%-5% doping levels.

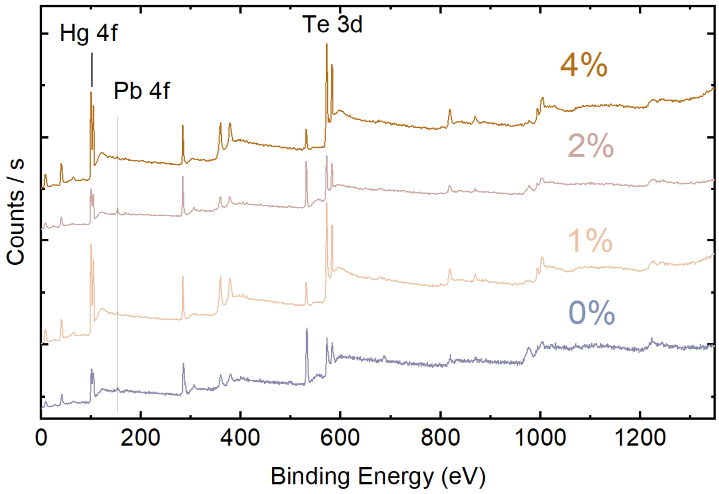

Figure S3. HgTe:Pb NPls survey XPS spectra.

Table S1. HgTe:Pb 1% sample XPS analysis

Name	Peak BE	FWHM eV	Area (P) CPS.eV	Atomic %	%
Hg	100.03	1.30	61103.35	9.48	55.8
Te	572.31	1.43	79394.76	7.43	43.7
Pb	138.14	1.20	675.72	0.07	0.4

Copyright: © 2022 by the authors. Licensee MDPI, Basel, Switzerland. This article is an open access article distributed under the terms and conditions of the Creative Commons Attribution (CC BY) license (https://creativecommons.org/licenses/by/4.0/).

Table S2. HgTe:Pb 2% sample XPS analysis

Name	Peak BE	FWHM eV	Area (P) CPS.eV	Atomic %	%
Hg	99.99	1.31	13279.20	3.28	52.5
Te	572.13	1.41	35164.65	2.93	46.9
Pb	138.51	1.98	489.31	0.04	0.6

Table S3. HgTe:Pb 4% sample XPS analysis

Name	Peak BE	FWHM eV	Area (P) CPS.eV	Atomic %	%
Hg	99.99	1.31	58543.97	9.89	54.4
Te	572.21	1.41	80031.34	8.15	44.9
Pb	138.17	1.57	1114.03	0.12	0.7

Photoluminescence decay measurement and analysis

A typical HgTe:Pb NPL PL decay curve recorded at 830 is demonstrated in Figure S4 below. Three-exponential fitting was achieved by reconvolution with recorded IRF.

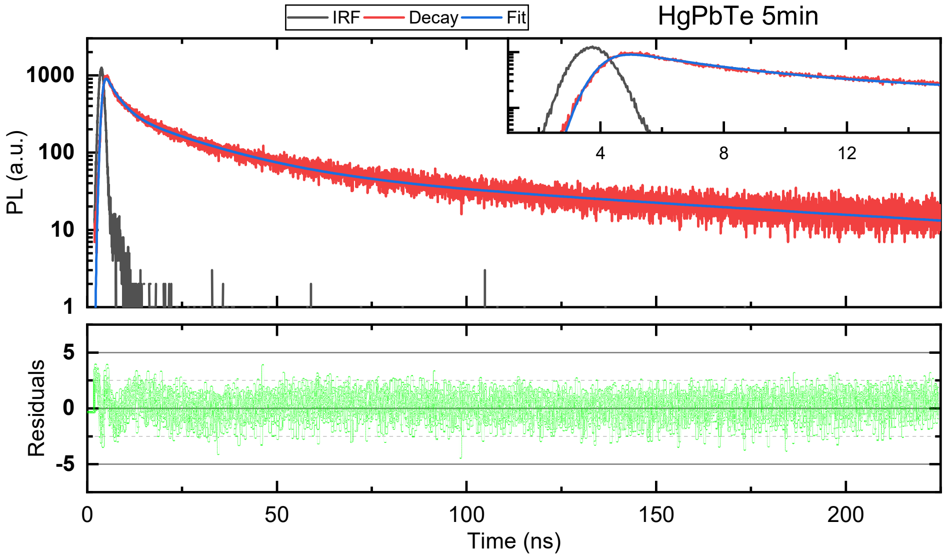

Figure S4. Typical HgPbTe (5 min reaction time) NPLs decay curve (red) taken at 830 nm and its 3-exponential fitting (blue) with IRF (black) and residuals (green).

The formulas used for the fitting and for the calculation of intensity-averaged decay time (τ_{Avg}) are as follows:

$$I(t) = A_0 + A_1\, exp\left(-\frac{t}{\tau_1}\right) + A_2\, exp\left(-\frac{t}{\tau_2}\right) + A_3\, exp\left(-\frac{t}{\tau_3}\right) \quad \text{(S1)}$$

$$\tau_{\mathrm{Avg}} = \frac{A_1\tau_1^2 + A_2\tau_2^2 + A_3\tau_3^2}{A_1\tau_1 + A_2\tau_2 + A_3\tau_3} \quad \text{(S2)}$$

where $A_i, i = 0, 1, 2, 3$ is the amplitude of the i-th component and τ_i is the respective decay time.

τ_{Avg}, calculated for 830 nm PL band, is demonstrated in Figure S5a. The largest contribution to the HgTe 830 nm PL band originates from the longest decay component A_3 with corresponding time constant τ_3; see Figure S5(b-d). τ_3 is significantly shorter for the pristine HgTe and HgTe:Pb with 1 minute reaction time; thus, averaged decay time is shorter for those samples. This might be the sign of some underlying physical processes, but we believe that this is a mere measurement error. From Figure S4, it is evident that recorded decay curves do not reach "zero" level during decay measurement time window. Hence, the data obtained for the longest decay component are not entirely reliable and the error in their determination should be high. Considering these facts, as well as the constant behavior of other time constants and amplitude components, we excluded the longest decay component from τ_{Avg} calculation. In this case, τ_{Avg} for 830 PL band, became the constant throughout the cation exchange reaction.

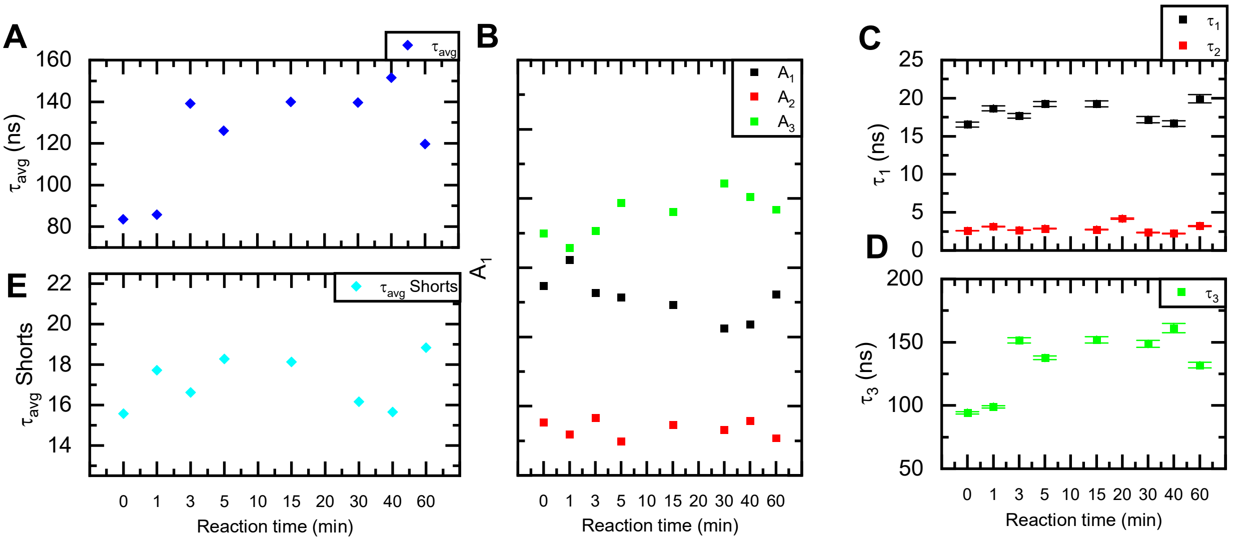

Figure S5. PL decay parameters for HgPbTe NPLs vs Pb exchange reaction time. (**A**) intensity-averaged decay time; individual components amplitudes (**B**) and times (**C–D**); (**E**) intensity-averaged decay time without the third component.

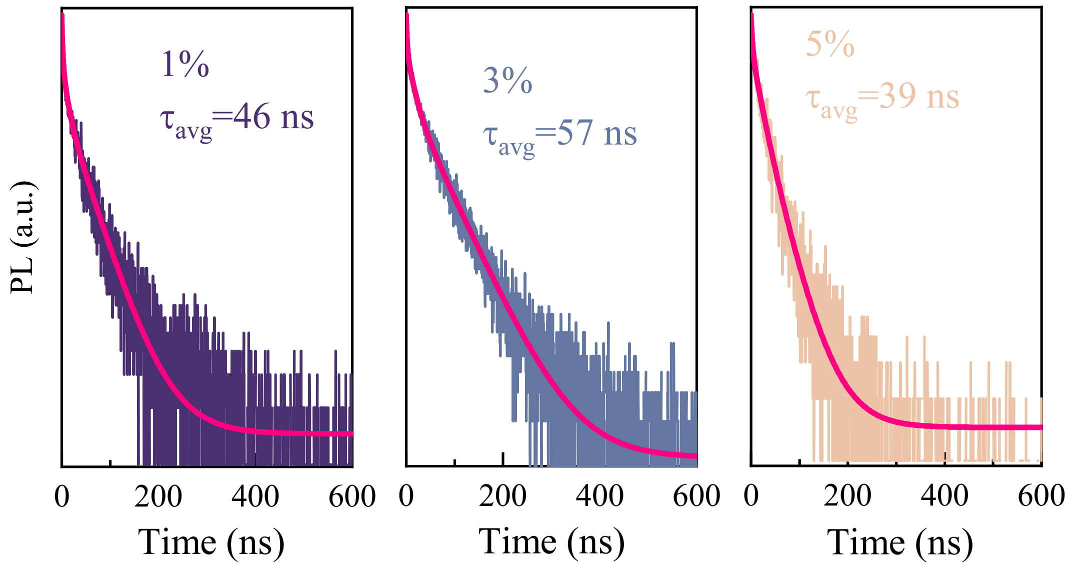

Figure S6. PL decay curves for NIR PL band of HgTe:Pb nanoplatelets.

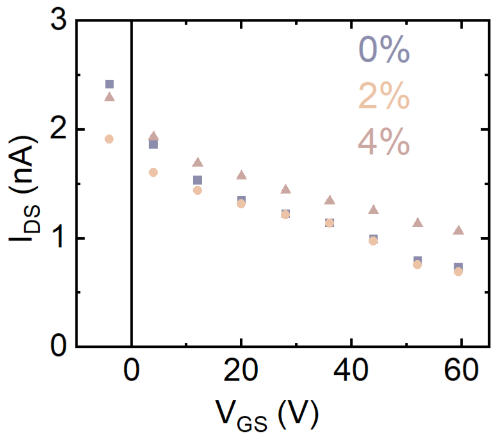

Figure S7. The n-channel transfer curves of a HgTe:Pb-EDT film samples.

References

1. Ithurria, S.; Tessier, M.D.; Mahler, B.; Lobo, R.P.S.M.; Dubertret, B.; Efros, Al.L. Colloidal Nanoplatelets with Two-Dimensional Electronic Structure. *Nat. Mater.* **2011**, 10, 936–941. https://doi.org/10.1038/nmat3145.
2. Izquierdo, E.; Robin, A.; Keuleyan, S.; Lequeux, N.; Lhuillier, E.; Ithurria, S. Strongly Confined HgTe 2D Nanoplatelets as Narrow Near-Infrared Emitters. *J. Am. Chem. Soc.* **2016**, 138, 10496–10501. https://doi.org/10.1021/jacs.6b04429.
3. Sun, H.; Buhro, W.E. Contrasting Ligand-Exchange Behavior of Wurtzite and Zinc-Blende Cadmium Telluride Nanoplatelets. *Chem. Mater.* **2021**, 33, 1683–1697. https://doi.org/10.1021/acs.chemmater.0c04247.
